# “*Where enjoyment begins, reengagement follows*” a higher-order and dimensional analysis of individual interest and study engagement within an integrated individual and dual sports physical education context

**DOI:** 10.3389/fspor.2026.1845127

**Published:** 2026-07-01

**Authors:** Joseph Lobo

**Affiliations:** College of Sports, Exercise and Recreation, Bulacan State University, Malolos, Philippines

**Keywords:** higher education, individual and dual sports, individual interest, physical education, study engagement

## Abstract

**Introduction:**

Student engagement in physical education (PE) is an important indicator of meaningful participation and learning in higher education. In the Philippines, where PE forms part of holistic student development, there is a need to understand how motivational constructs operate within specific activity contexts. This study examined the association between individual interest and study engagement among Filipino college students enrolled in an integrated PE context involving individual and dual sports.

**Methods:**

A quantitative cross-sectional design was used with 327 undergraduate students enrolled in PATH-Fit 4 at a state university in the Philippines. Drawing on the tripartite model of individual interest, it was modeled as a reflective-reflective higher-order construct composed of positive affect and willingness to reengage (PAWR), stored utility value (SUV), and stored attainment value and knowledge-seeking intentions (SAVKSI). Data were analyzed using partial least squares structural equation modeling.

**Results:**

The higher-order model showed that individual interest was positively and significantly associated with study engagement, although the effect size and explanatory power were small. The dimensional model explained a greater proportion of variance than the higher-order model. Among the three factors, only PAWR demonstrated a significant positive association with study engagement, while SUV and SAVKSI were not significant. These findings indicate that the dimensions of individual interest were not equally associated with engagement in this context.

**Discussion:**

The results suggest that individual interest is a coherent multidimensional construct, but its engagement relevance depends on the specific factors examined. In PE involving individual and dual sports, affective attraction and willingness to reengage appear to be more immediately associated with students' engagement than perceived usefulness or knowledge-seeking orientation. The findings provide preliminary support for a context-sensitive interpretation of the tripartite model and highlight the importance of designing sport-based PE experiences that foreground enjoyment, emotional safety, manageable challenge, and opportunities for repeated participation.

## Introduction

Students encounter a variety of sport formats during physical education (PE) lessons, including activities that are performed individually as well as those involving direct competition with a single opponent (1). In many PE curricula, such as in Philippine higher education (HE), individual and dual sports are introduced within the same instructional units rather than as entirely separate learning tracks (2, 3). This curricular arrangement allows students to experience both personal skill development and interactive forms of play within a shared learning environment (4). Although these activities share common instructional goals, they expose students to distinct but complementary participation demands during PE lessons (5). Individual sports typically emphasize self-paced performance and personal mastery of movement skills (6), whereas dual sports involve direct exchanges with an opponent that require reactive decision-making and strategic responses (7). These activities form an integrated sport-based PE context in which students encounter both self-referenced challenge and opponent-responsive participation. Despite their frequent inclusion within the same curricular setting, relatively little attention has been given to how motivational orientations relate to students' engagement in this combined individual and dual sports learning context.

Study engagement has become an important construct for understanding how students invest effort, attention and commitment in learning activities (8). Conceptualized as a positive and fulfilling state characterized by vigor, dedication and absorption, engagement reflects the extent to which students are energetically involved in academic tasks and psychologically immersed in the learning process (9). In PE, engagement extends beyond mere physical participation (10); it represents the degree to which students actively involve themselves in movement experiences and skill development (11). Students who demonstrate higher levels of engagement often exhibit persistence during challenging tasks, enthusiasm toward participation and sustained attention during practice and gameplay (12, 13). However, engagement in PE does not occur uniformly across all activities (12). Previously conducted studies observed that students frequently demonstrate varying levels of enthusiasm and commitment depending on the nature of the sports or movement tasks they perform (14, 15). Hence, this suggests that motivational orientations may be associated with how students involve themselves in learning experiences.

One motivational construct that has received increasing attention in educational and sport pedagogy research is individual interest (16). Individual interest refers to a relatively enduring predisposition to engage with particular activities that individuals perceive as enjoyable, meaningful or personally valuable (17). Unlike situational interest, which arises temporarily from environmental stimuli (18), individual interest develops gradually through repeated positive experiences and becomes a stable orientation toward specific activities (19). In PE contexts, students who develop strong interest in certain sports or movement activities often demonstrate sustained participation, persistence during challenging tasks and greater enjoyment of learning experiences (16). Therefore, interest represents an important motivational orientation associated with students' willingness to remain engaged in movement-based learning activities during PE lessons (20, 21).

To better understand the multidimensional nature of individual interest in PE, Roure et al. (22) proposed the tripartite model of individual interest, which conceptualizes interest through three interrelated factors: positive affect and willingness to reengage (PAWR), stored utility value (SUV), and stored attainment value and knowledge-seeking intentions (SAVKSI). The PAWR factor reflects the affective enjoyment students experience when participating in an activity and their inclination to reengage with similar experiences in the future (22, 23). SUV represents the perceived usefulness or practical value that students associate with the activity (22, 23), while SAVKSI captures the personal importance of the activity and students' motivation to acquire additional knowledge and skills related to it (22, 23). Together, these factors provide a multidimensional framework for understanding how emotional enjoyment, perceived value and personal meaning correspond with students' engagement in learning activities. Although the tripartite model has been examined in several PE contexts, its potential for explaining engagement across different sport participation formats remains insufficiently explored.

Although individual and dual sports are frequently taught within the same PE curricula in Philippine HE, their combined instructional arrangement creates a distinctive sport-based learning context. Individual sports often emphasize personal mastery and self-directed performance (24), whereas dual sports involve interactive exchanges with an opponent that require immediate strategic responses (25). In PATH-Fit classes, however, these activities are commonly encountered as part of one integrated learning experience rather than as separate analytic groups. This means that students' engagement may be shaped by their overall experience of a PE context that combines personal challenge, skill execution, immediate feedback and interactive play. However, empirical research examining how the factors of individual interest are associated with engagement in this integrated context remains limited. Therefore, this study examines whether the tripartite model of individual interest is associated with study engagement among students participating in individual and dual sports in PE.

### Sport structures of individual and dual sports in physical education

PE curricula in the HE commonly organize sport activities into categories based on participation structure, including individual, dual and team sports (26). This classification is widely used in sport pedagogy because it reflects how interaction occurs during performance and learning tasks (27). Individual sports generally involve activities in which participants perform independently and focus on improving their own performance outcomes (28). In these activities, students typically concentrate on refining movement techniques, developing personal mastery and monitoring their own progress during practice or competition (29). Examples frequently introduced in school settings include athletics events and certain forms of fitness-based skill development. These activities often emphasize self-regulation and personal responsibility during participation, allowing students to engage with tasks in a manner that highlights individual skill refinement and personal achievement.

On the other hand, dual sports involve direct competition between two players who interact continuously during gameplay (30). Activities such as badminton, tennis and table tennis are commonly categorized as dual sports because participation requires players to respond to an opponent's actions while simultaneously executing their own strategies (31). In these activities, the learning experience often involves reactive decision-making, tactical exchanges and the anticipation of an opponent's movement patterns. Unlike team sports that involve cooperation among multiple players, dual sports emphasize one-on-one interaction while still requiring players to adapt their performance according to the behavior of another participant (32). These characteristics create a distinct participation dynamic in which students engage with both the task itself and the actions of their opponent.

Despite these structural distinctions, individual and dual sports frequently appear together within PE curricula in the HE in the Philippines because they share several pedagogical objectives. Both formats provide opportunities for skill acquisition, physical conditioning and the development of tactical awareness during gameplay. PE teachers often incorporate these activities within the same instructional units to expose students to varied movement experiences and participation formats (33). Through these experiences, students encounter both self-directed performance tasks and interactive competitive situations, which may correspond with different psychological responses during participation (34).

Given that students often engage with both individual and dual sports within the same learning environment, examining how motivational orientations relate to engagement in this integrated context may provide useful insights for PE pedagogy. In the present study, individual and dual sports are approached as a shared PATH-Fit learning context where students encounter varied movement demands, self-directed performance tasks and interactive competitive situations. Understanding how students experience and involve themselves in these commonly taught activities may contribute to a more nuanced perspective on engagement within movement-based learning contexts.

### Study engagement in physical education

Study engagement has received considerable attention in educational psychology as a construct describing students' active involvement in learning activities (35). Engagement is commonly conceptualized as a positive and fulfilling state characterized by vigor, dedication and absorption (36). Vigor refers to the energy and persistence students demonstrate during learning tasks (37), dedication reflects their sense of enthusiasm and commitment toward academic activities (38), and absorption represents the degree to which students become deeply immersed in what they are doing (39). Together, these dimensions capture the extent to which students invest effort, attention and psychological involvement in educational experiences (40). In educational research, these dimensions are frequently operationalized using the Utrecht Work Engagement Scale for Students (UWES-9S) developed by Carmona-Halty et al. (41), a widely used instrument designed to measure students' engagement in academic contexts. In educational contexts, higher levels of engagement are often associated with greater persistence in learning tasks and stronger involvement in academic activities (41).

In PE, engagement extends beyond simple physical participation (11). Because PE lessons involve movement-based learning, students are expected not only to perform physical tasks but also to remain mentally attentive to skill execution, tactical awareness and instructional feedback (42). Engaged students tend to demonstrate active participation during drills and gameplay, sustained concentration during skill practice, and continued effort when encountering challenging physical tasks. This form of engagement reflects a combination of physical involvement and psychological immersion in the learning experience, highlighting the importance of examining how students invest themselves in movement-based activities (43, 44).

Research in sport pedagogy has increasingly emphasized engagement as an important indicator of meaningful participation in PE (12, 45). Students who report higher engagement during PE lessons often demonstrate greater enthusiasm toward participation, stronger commitment to learning activities and increased willingness to invest effort during practice (46, 47). Engagement may also correspond with how students perceive the learning environment, the nature of the activities being performed and the personal relevance they attribute to movement experiences (48–50). These factors suggest that engagement in PE is closely connected to students’ motivational orientations toward specific sports or movement activities.

Despite its importance, engagement in PE is not experienced uniformly across all activities (12). Students may demonstrate varying levels of energy, dedication and immersion depending on the types of sports or movement tasks they encounter during lessons. Activities that resonate with students' interests or personal preferences may correspond with greater psychological involvement (51), while less appealing activities may be associated with reduced participation or attentiveness (52). Consequently, examining the motivational factors related to study engagement may provide valuable insights into how students experience and participate in PE learning environments.

### Individual interest in physical education

Interest has long been recognized as an important motivational construct in educational psychology, particularly in relation to students' engagement in learning activities (21). Among the different forms of interest, individual interest refers to a relatively enduring predisposition to engage with particular topics, activities or domains that individuals perceive as enjoyable, meaningful or personally valuable (53). Unlike situational interest, which arises temporarily from features of the immediate learning environment (54), individual interest develops gradually through repeated experiences and sustained involvement with specific activities (55). This developmental process has been described in the Four-Phase Model of Interest (56, 57), which outlines how interest evolves from triggered situational interest to maintained situational interest, emerging individual interest, and eventually well-developed individual interest. According to this model, repeated exposure to meaningful learning experiences may support the gradual formation of stable motivational orientations toward specific activities (56). Over time, these experiences contribute to the formation of stable preferences and motivational orientations toward certain learning tasks (58).

Within Physical Education, individual interest is often associated with how students respond to different sports or movement activities encountered during lessons (59). Students frequently demonstrate varying degrees of enthusiasm toward particular activities depending on their prior experiences, perceived competence and personal preferences (60). When students develop interest in certain sports, they tend to participate more actively during lessons, demonstrate greater willingness to practice skills, and show sustained involvement during gameplay and movement tasks (61). Therefore, individual interest represents an important motivational orientation that corresponds with students' willingness to participate and remain engaged in physical activities (62).

The development of individual interest in PE may also be related to how students perceive the personal value and enjoyment associated with movement experiences (55). Activities that students find enjoyable, meaningful or relevant to their personal goals are more likely to attract sustained attention and continued participation (63). Through repeated engagement with these activities, students may develop deeper connections with particular sports or movement practices, strengthening their inclination to reengage with similar experiences in the future (64). These processes highlight the role of individual interest as a motivational orientation that accompanies students' ongoing participation in these types learning environments.

Given its association with participation and involvement in learning activities, individual interest has been increasingly examined within PE research (65, 66). Scholars have explored how students' enduring preferences for particular sports or activities relate to their participation patterns, enjoyment of movement experiences and willingness to remain involved in lessons (67). These investigations suggest that individual interest represents a meaningful motivational construct for understanding how students approach movement-based learning activities. Building on this perspective, recent theoretical developments have sought to conceptualize individual interest in PE through multidimensional frameworks that capture both affective and cognitive aspects of interest (22).

### The tripartite model of individual interest

To provide a multidimensional perspective on how individual interest operates in PE, Roure et al. (22) proposed the tripartite model of individual interest, which conceptualizes interest through three interrelated factors: positive affect and willingness to reengage (PAWR), stored utility value (SUV), and stored attainment value and knowledge-seeking intentions (SAVKSI). This framework extends earlier conceptualizations of interest by integrating affective experiences, perceived usefulness and personal significance as components of sustained motivational orientation in PE (22). PAWR represents the emotional component of interest, reflecting students' enjoyment of an activity and their willingness to return to similar experiences in the future (23, 64). This factor resonates with Self-Determination Theory, which emphasizes intrinsic motivation and positive emotional experience as important energizing conditions for sustained engagement (68, 69). Meanwhile, SUV reflects the perceived usefulness or practical relevance of an activity, including its contribution to fitness, skill development or broader personal goals (23, 70, 71). Finally, SAVKSI captures the personal importance students attribute to an activity and their inclination to deepen their knowledge or competence in it (23, 71). Both SUV and SAVKSI are conceptually aligned with Expectancy-Value Theory, particularly utility value and attainment value, because they reflect how students evaluate the usefulness, personal relevance and importance of participation (72, 73).

Thus, the three factors provide a multidimensional representation of individual interest that captures both affective and cognitive aspects of students' motivational orientations toward movement activities. Importantly, the tripartite model has also received local psychometric support in the Philippines, suggesting that its three-dimensional structure can be meaningfully applied in this educational and cultural context (74). This local validation provides a stronger basis for using the model beyond its original development context and supports its relevance for examining Filipino students' interest in PE activities. Building on this theoretical and measurement foundation, the present study adopts the tripartite model not only to determine whether individual interest is associated with study engagement, but also to examine whether its factors carry similar or differentiated relevance within the integrated PE context involving individual and dual sports.

### Linking individual interest and study engagement in physical education

A growing body of research in educational psychology suggests that students' interest in learning activities is closely associated with their engagement during academic tasks (16). Interest represents a motivational orientation that encourages students to invest attention, effort and persistence in learning experiences (19). When students perceive activities as enjoyable, meaningful or personally valuable, they are more likely to participate actively and remain psychologically involved in learning tasks (36). This relationship has been widely documented across different academic domains (75), indicating that students' enduring preferences for particular activities may correspond with their willingness to remain involved in learning processes. In PE, this connection is especially important because engagement is expressed through embodied participation, sustained effort, skill practice and psychological immersion in movement-based tasks. Recent applications of the tripartite model in Philippine PE contexts further suggest that the affective, utility-based and attainment-oriented factors of individual interest may not operate with the same salience across all movement settings, but may vary depending on the nature of the activity, the learning demands involved and the meanings students attach to participation (59, 64, 76–82).

Despite these developments, relatively little research has examined how the factors of individual interest relate to study engagement within integrated sport-based PE contexts. Individual and dual sports are commonly included within PE curricula, yet they are often taught as part of a shared learning unit in which students experience both self-referenced and opponent-responsive movement tasks. This combined context may activate distinct motivational processes because students must negotiate personal challenge, skill execution, immediate feedback, competitive interaction and repeated participation within the same learning environment. Understanding how students' motivational orientations relate to engagement in this context may therefore provide useful insights for PE pedagogy. Building on the tripartite model of individual interest and prior PE studies in the Philippines showing the context-sensitive functioning of interest factors, the present study examines whether individual interest, both as a higher-order construct (HOC) and through its factors, is associated with study engagement among students participating in individual and dual sports in PE.

### Objectives of the study and hypotheses formulation

Building on literature suggesting that motivational orientations are associated with students’ involvement in learning activities, the present study examines the relationship between individual interest and study engagement in PE within the context of individual and dual sports in Philippine HE. Drawing on the tripartite model of individual interest proposed by Roure et al. (22), this study conceptualizes individual interest as a multidimensional construct composed of PAWR, SUV, and SAVKSI. Rather than treating individual interest only as a general motivational disposition, the study examines whether its factors carry similar or differentiated relevance for engagement in an integrated sport-based PE context involving individual and dual sports. This focus responds to the need for a more context-sensitive understanding of how affective, utility-based and attainment-oriented components of interest operate in movement-based learning environments.

The study is structured in two phases. First, individual interest is modeled as a reflective-reflective HOC to examine its overall association with study engagement. Second, an additional analysis is conducted at the dimensional level to explore the relative associations of PAWR, SUV and SAVKSI with study engagement. This dual approach allows for the examination of both the structural coherence of individual interest and the differentiated roles of its factors. In doing so, the study contributes to the theoretical refinement of the tripartite model by clarifying whether engagement in individual and dual sports is associated with individual interest as a unified construct, or whether specific factors of interest are more salient than others. Specifically, the study examines the association between individual interest and study engagement, as well as the separate associations of PAWR, SUV and SAVKSI with study engagement in PE.

## Methods

### Research design and participants

This study employed a quantitative cross-sectional survey design to examine the association between individual interest and study engagement in the context of individual and dual sports within PE classes. A total of 327 undergraduate students enrolled in Physical Education courses, specifically PATH-Fit 4: Physical Activity Towards Health and Fitness 4, participated in the study. The course included both individual and dual sports activities and was offered at a state university in the Philippines during the Second Semester of Academic Year 2025–2026. Participants were selected through purposive sampling based on their enrollment in PATH-Fit 4 classes that incorporated individual and dual sports within the same instructional units. Data were collected through a self-administered online survey during the final weeks of the semester (March to April 2026), when students had already been exposed to both sport formats as part of their coursework.

In the present study, individual and dual sports were treated as one integrated PATH-Fit learning context rather than as separate instructional or analytic groups. This reflects the actual curricular arrangement of PATH-Fit 4, where students encounter individual and dual sports within the same course experience. Accordingly, participants responded to the survey based on their overall experience of the course context involving individual and dual sports, rather than on a single sport type or a separately assigned sport modality. Because the dataset did not include separate grouping information indicating whether each response was anchored exclusively in an individual sport or a dual sport, sport modality was not modeled as a moderator and no split-sample comparison was conducted.

### Instruments

Data were collected using a structured online survey administered via Google Forms, with access provided through the official learning management systems of the participating classes. The questionnaire consisted of three sections. The first section collected demographic information, including age and sex. Individual interest was measured using the Students' Individual Interest in Physical Education Questionnaire developed by Roure et al. (22). The instrument assesses three factors: positive affect and willingness to reengage (PAWR), stored utility value (SUV), and stored attainment value and knowledge-seeking intentions (SAVKSI). The questionnaire consists of 14 items rated on a 5-point Likert scale ranging from 1 (strongly disagree) to 5 (strongly agree). Minor contextual wording adjustments were made to align the items with individual and dual sports activities while preserving their original conceptual meaning (e.g., “physical activities” to “individual and dual sports”). Study engagement was measured using the Utrecht Work Engagement Scale for Students (UWES-9S) developed by Carmona-Halty et al. (41). The instrument contains nine items assessing three dimensions of engagement: vigor, dedication and absorption, rated on a 7-point Likert scale ranging from 0 (Never) to 6 (Always). A composite score was computed to represent overall study engagement. Participation was anonymous, and instructors who disseminated the survey link had no access to information regarding who responded, ensuring the voluntary nature of participation.

#### Evaluation of measurement model and convergent validity

The measurement model was evaluated in terms of indicator reliability, internal consistency reliability, convergent validity and collinearity diagnostics. As shown in Table 1, all indicators exhibited satisfactory loadings (≥0.70), thereby confirming adequate indicator reliability. Internal consistency reliability was established, with Cronbach's alpha (CA) and composite reliability (CR) values for all constructs exceeding the acceptable threshold (≥0.70). Specifically, CA values ranged from 0.830 to 0.935, while CR values ranged from 0.890 to 0.947, indicating a high level of internal consistency across constructs. Convergent validity was also supported, as the average variance extracted (AVE) values for all constructs exceeded the recommended minimum (≥0.50), ranging from 0.685 to 0.789. In addition, collinearity diagnostics indicated no multicollinearity concerns, with all outer variance inflation factor (VIF) values below the threshold (<5). Overall, these findings demonstrate that the measurement model exhibits satisfactory reliability and validity, supporting its suitability for subsequent structural model and HOC evaluation.

**Table 1:** Measurement model assessment.

Constructs	Items	Item loadings	CA	CR	AVE	VIF (Outer model)
PAWR	PAWR2	0.902	0.866	0.896	0.685	2.094
	PAWR3	0.745				2.171
	PAWR4	0.890				2.050
	PAWR5	0.760				2.111
SUV	SUV1	0.895	0.830	0.890	0.730	1.717
	SUV3	0.848				1.973
	SUV4	0.819				2.261
SAVKSI	SAVKSI1	0.867	0.916	0.937	0.789	2.968
	SAVKSI2	0.867				3.650
	SAVKSI3	0.895				2.767
	SAVKSI5	0.922				2.837
UWES	VIG2	0.871	0.935	0.947	0.718	2.972
	VIG3	0.856				3.521
	DED2	0.798				2.920
	DED3	0.852				3.912
	ABS1	0.864				3.459
	ABS2	0.881				3.835
	ABS3	0.804				2.432

Item loadings ≥ 0.70, Cronbach's Alpha (CA) and Composite Reliability (CR) ≥ 0.70, AVE (Average Variance Extracted) ≥ 0.50, VIF (Variance Inflation Factor-Outer Model) < 5.0.

PAWR, positive affect and willingness to reengage; SUV, stored-utility value; SAVKSI, stored attainment value and knowledge-seeking intentions; UWES, study engagement; VIG, vigor; DED, dedication; ABS, absorption.

#### Discriminant validity

Discriminant validity was assessed using both the Fornell-Larcker criterion and the Heterotrait-Monotrait ratio (HTMT). As presented in Table 2, the square roots of the average variance extracted (√AVE) for each construct exceeded the corresponding inter-construct correlations, thereby satisfying the Fornell-Larcker criterion and supporting adequate discriminant validity. With respect to HTMT, most values were below the conservative threshold (≤0.85), indicating that the constructs are empirically distinct. However, the HTMT value between PAWR and SAVKSI slightly exceeded the conservative cutoff but remained below the more liberal threshold (≤0.90). This suggests that while these constructs are closely related, they still demonstrate acceptable discriminant validity.

**Table 2:** Discriminant validity assessment using fornell-larcker criterion and HTMT.

Fornell-larcker criterion				
	PAWR	SAVKSI	UWES	SUV
PAWR	0.827			
SAVKSI	0.742	0.888		
UWES	0.243	0.199	0.847	
SUV	0.615	0.733	0.087	0.855
Heterotrait-monotrait ratio (HTMT)				
PAWR				
SAVKSI	0.866			
UWES	0.213	0.178		
SUV	0.789	0.837	0.097	

Diagonal elements represent the square root of the average variance extracted (√AVE). HTMT ratio value ≤ 0.85 (conservative) ≤ 0.90 (liberal) approach.

PAWR, positive affect and willingness to reengage; SUV, stored-utility value; SAVKSI, stored attainment value and knowledge-seeking intentions; UWES, study engagement.

### Data analysis

A two-step analytical approach was employed using Partial Least Squares Structural Equation Modeling (PLS-SEM). PLS-SEM was selected due to its suitability for modeling latent constructs and its flexibility in handling complex models without strict distributional assumptions. First, the measurement model was evaluated to assess indicator reliability, internal consistency reliability, convergent validity and discriminant validity. Second, the structural model was assessed to examine the associations among constructs. Individual interest was modeled as a reflective-reflective HOC using the repeated indicators approach, wherein all indicators of the LOC were assigned to the HOC. To further examine the relative contribution of each factor of individual interest, an additional structural model was estimated in which PAWR, SUV and SAVKSI were directly specified as predictors of study engagement. This supplementary analysis enabled the comparison between higher-order and dimensional models in terms of their associations with the outcome variable. All analyses were conducted using SmartPLS version 4.1.1.8.

To address potential concerns related to common method variance, both procedural and statistical precautions were considered. Procedurally, the survey was administered anonymously, and instructors who distributed the survey link had no access to information regarding which students participated. Statistically, collinearity diagnostics were examined through outer and inner VIF values. Although VIF analysis does not eliminate the possibility of common method variance, the absence of problematic collinearity provides supplementary evidence that the estimated relationships were not severely inflated by common-method-related bias. However, this diagnostic should be interpreted cautiously because it does not substitute for stronger procedural remedies such as temporal separation, multi-source data, or the inclusion of objective engagement indicators.

### Ethical considerations

This study was approved by the Bulacan State University Ethics Research Committee (BulSU-ERC) under Protocol Code BulSUERC-2026-0080-01. Participation was voluntary, and informed consent was obtained from all participants prior to data collection. The study complied with applicable ethical standards and the provisions of the Philippine Data Privacy Act of 2012.

## Results

Collinearity among the predictor constructs was assessed using the variance inflation factor (VIF). As presented in Table 3, all inner VIF values ranged from 2.216 to 3.068, which are below the recommended threshold (<5.00). This indicates that multicollinearity is not a concern in the structural model. In addition, the VIF values were below the more conservative (<3.3) guideline commonly used as a collinearity-based diagnostic for possible common-method-related inflation. Although this does not rule out common method variance, the results do not indicate substantial collinearity or severe common-method-related distortion in the structural estimates. These findings should therefore be interpreted as supplementary evidence supporting the stability of the structural model, rather than as definitive evidence that common method variance was absent.

**Table 3:** VIF of the inner model.

Inner model	UWES
PAWR	2.282
SUV	2.216
SAVKSI	3.068

VIF (Variance Inflation Factor-Inner Model) < 5.0.

PAWR, positive affect and willingness to reengage; SUV, stored-utility value; SAVKSI, stored attainment value and knowledge-seeking intentions; UWES, study engagement.

Prior to interpreting the structural model, the reflective-reflective HOC evaluation was conducted to assess whether the three factors functioned as reflective LOCs of INDINT. As shown in Table 4, all LOCs loaded strongly and significantly on INDINT. SAVKSI showed the highest loading (λ = .938, SE = .007, *t* = 129.828, *p* < .001, 95% CI [.923, .951), followed by PAWR (λ = .909, SE = .015, *t* = 58.969, *p* < .001, 95% CI [.875, .935) and SUV (λ = .862, SE = .019, *t* = 44.241, *p* < .001, 95% CI [.818, .894). The CI for all three loadings did not include zero, further supporting the stability of the estimates. These findings support the specification of INDINT as a reflective-reflective multidimensional construct in PE involving individual and dual sports. The strong loadings indicate that the three factors each contributed substantially to the HOC, suggesting that INDINT in this context is composed of affective, utility-based and attainment-oriented components. Although SAVKSI demonstrated the strongest loading, the consistently high loadings across all three factors indicate that INDINT operates as a coherent motivational construct rather than as a single-dimension representation of students' interest. This provides support for proceeding to the structural model, where INDINT was examined in relation to UWES.

**Table 4:** Higher-order construct evaluation of individual interest.

HOC	LOC	Loading λ	SE	*t*	*p*	95% CI [LL, UL]
INDINT	PAWR	0.909	0.015	58.969	<.001	[0.875, 0.935]
	SUV	0.862	0.019	44.241	<.001	[0.818, 0.894]
	SAVKSI	0.938	0.007	129.828	<.001	[0.923, 0.951]

HOC, higher-order construct; LOC, lower-order construct; SE, standard error; CI, confidence interval; LL, lower limit; UL, upper limit. The values represent standardized reflective loadings of the lower-order constructs on the higher-order construct. All loadings were statistically significant at *p* < .001.

INDINT, individual interest; PAWR, positive affect and willingness to reengage; SUV, stored utility value; SAVKSI, stored attainment value and knowledge-seeking intention.

### Structural model assessment

**Figure 1:**
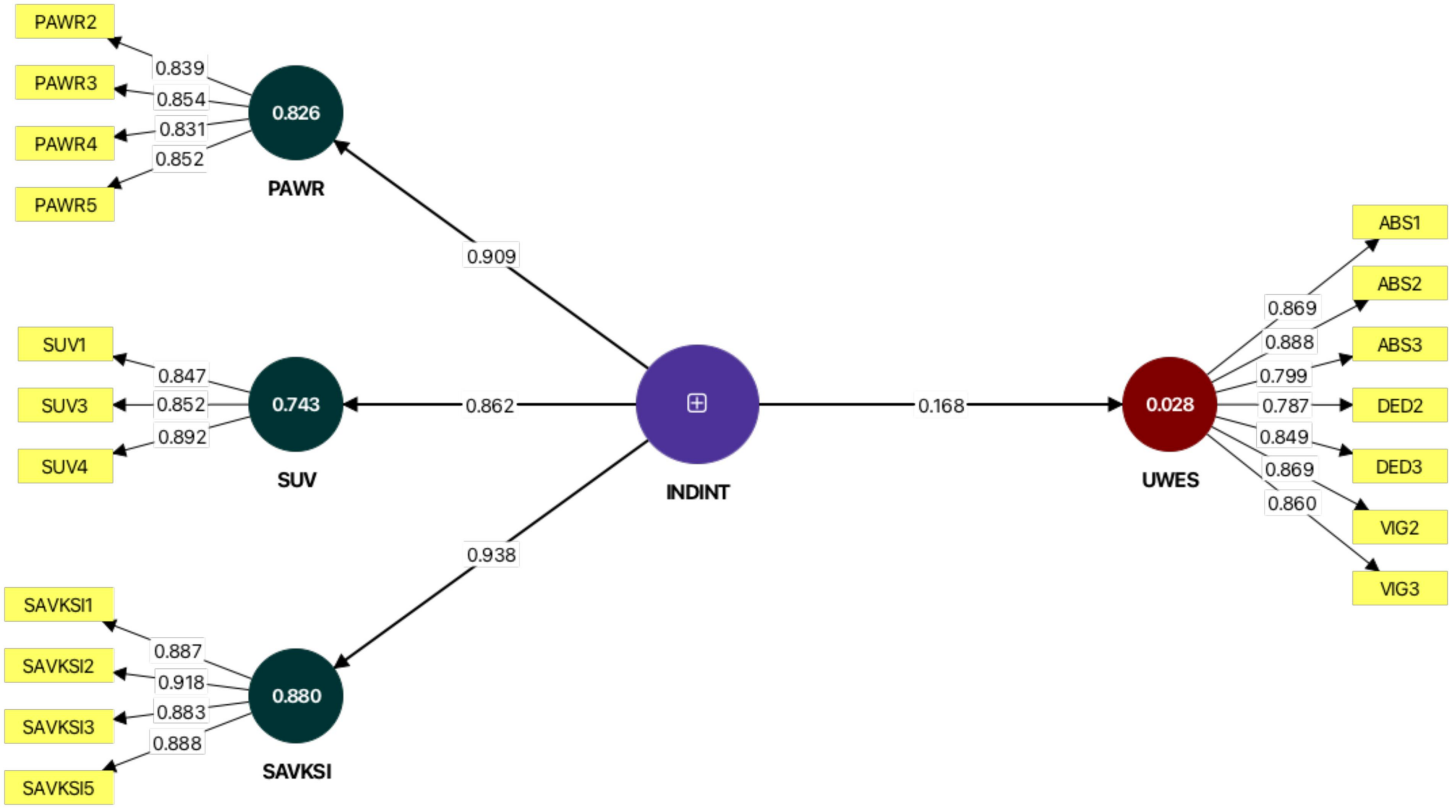
Higher-order structural model of individual interest and study engagement. The model presents individual interest as a reflective-reflective higher-order construct composed of PAWR, SUV and SAVKSI. Path coefficients are shown alongside *R*^2^ values for the endogenous construct.

The explanatory power of the structural model was assessed using the coefficient of determination (*R*^2^) of the endogenous construct. In structural equation modeling, *R*^2^ values represent the proportion of variance in the dependent variable explained by its predictor(s). As shown in Figure 1, the model explained 2.8% of the variance in study engagement (*R*^2^ = .028), indicating a small level of explanatory power. This suggests that individual interest provides a preliminary motivational explanation of study engagement in this context, but its explanatory contribution is limited. Accordingly, the model should be interpreted as identifying one motivational correlate of engagement rather than as providing a comprehensive account of the factors that shape students' engagement in PE.

The structural model was evaluated by examining the path relationship between INDINT and UWES. As presented in Table 5, INDINT demonstrated a positive and statistically significant association with UWES (*β* = .168, SE = .078, *t* = 2.159, *p* = .031, 95% CI [.067, .324). The confidence interval did not include zero, further supporting the stability of the estimated relationship. However, the effect size was small (*f*^2^ = .029), indicating that although INDINT was significantly associated with UWES, its practical explanatory contribution was limited. This suggests that students with higher levels of INDINT in PE involving individual and dual sports tend to report higher levels of UWES, but the strength of this relationship is small. The finding indicates that individual interest functions as a meaningful motivational correlate of engagement, particularly when students perceive the learning experience as enjoyable, valuable or personally relevant. At the same time, the small effect size suggests that engagement in individual and dual sports is multidetermined and likely shaped by other instructional, social, contextual and learner-related factors beyond individual interest alone. Thus, INDINT appears to contribute to students' energy, dedication and absorption in PE, but it should be understood as one component within a broader ecology of engagement rather than as a singular or exhaustive explanation. Therefore, the proposed association between INDINT and UWES was supported.

**Table 5:** Structural model results.

Path	*β*	SE	*t-*value	*p*-value	*f* ^2^	95% CI [LL, UL]	Decision
INDINT → UWES	0.168	0.078	2.159	0.031	0.029	[0.067, 0.324]	Supported

*β*, standardized path coefficient; SE, standard error; *f*^2^, effect size; CI, confidence interval; LL, lower limit; UL, upper limit. Path significance was determined using bootstrapping with 10,000 subsamples, while *f*^2^ was obtained from the PLS Algorithm results.

INDINT, individual interest; UWES, study engagement.

### Additional analysis of dimensional contributions to study engagement

To further examine the factor-specific contribution of INDINT, an additional structural model was estimated in which PAWR, SUV and SAVKSI were directly specified as predictors of UWES within the context of individual and dual sports. As shown in Table 6, the model explained 7.2% of the variance in UWES (*R*^2^ = .072), which was higher than the explanatory power observed in the higher-order model (*R*^2^ = .028), although still small in magnitude. This suggests that examining the factors separately provided a more specific account of how INDINT relates to UWES in this integrated sport-based PE context. Among the three factors, only PAWR demonstrated a positive and statistically significant association with UWES (*β* = .237, SE = .109, *t* = 2.169, *p* = .030, 95% CI [.004, .430). The CI did not include zero, further supporting the statistical stability of the estimated relationship, although the lower bound was close to zero and should be interpreted with caution. The effect size for PAWR was small (*f*^2^ = .027), indicating that its distinct practical contribution was limited but meaningful within the model. In contrast, SUV did not demonstrate a statistically significant association with UWES [*β* = −.171, SE = .118, *t* = 1.450, *p* = .147, 95% CI (−.325, .147), *f*^2^ = .014]. Because the CI included zero, this path was not supported. Although the coefficient was negative, the non-significant result indicates that it should not be interpreted as evidence of an inverse relationship. Rather, the finding suggests that perceived usefulness did not make a distinct contribution to UWES when examined alongside the affective and attainment-related factors of INDINT. Similarly, SAVKSI was not significantly associated with UWES [*β* = .151, SE = .128, *t* = 1.180, *p* = .238, 95% CI (−.159, .337), *f*^2^ = .008]. The CI also included zero, indicating that this path was not supported. Thus, students' attainment value and knowledge-seeking intentions were not distinctly associated with engagement in this model. In this regard, the findings suggest that the factors of INDINT were not equally associated with UWES in PE involving individual and dual sports. More specifically, PAWR emerged as the only factor with a distinct positive association, indicating that engagement in this context may be more closely linked to students' enjoyment, positive affect and willingness to reengage than to perceived usefulness or knowledge-seeking orientation alone. This pattern helps explain why the higher-order model produced a small association, because combining factors with different levels of relevance may reduce predictive specificity. Thus, the dimensional analysis provides a more nuanced interpretation by showing that the affective component of INDINT appears to carry the strongest engagement-related signal in this integrated sport-based PE context.

**Table 6:** Additional structural model results.

Path	*β*	SE	*t-*value	*p*-value	*f* ^2^	95% CI [LL, UL]	Decision
PAWR → UWES	0.237	0.109	2.169	0.030	0.027	[0.004, 0.430]	Supported
SUV → UWES	−0.171	0.118	1.450	0.147	0.014	[−0.325, 0.147]	Not supported
SAVKSI → UWES	0.151	0.128	1.180	0.238	0.008	[−0.159, 0.337]	Not supported

*β*, standardized path coefficient; SE, standard error; *f*^2^, effect size; CI, confidence interval; LL, lower limit; UL, upper limit. Path significance was determined using bootstrapping with 10,000 subsamples, while *f*^2^ values were obtained from the PLS Algorithm results.

PAWR, positive affect and willingness to reengage; SUV, stored utility value; SAVKSI, stored attainment value and knowledge-seeking intention; UWES, study engagement.

## Discussion

The present study examined the association between individual interest and study engagement within an integrated PE context involving individual and dual sports in Philippine HE, while also modeling individual interest as a HOC. The findings provide preliminary evidence that individual interest is structurally coherent but only weakly associated with study engagement when modeled as a HOC. At the dimensional level, the findings further suggest that the factors of individual interest do not carry equal engagement relevance. Rather than presenting individual interest as a strong or exhaustive explanation of engagement, the study positions it as one motivational component within a broader ecology of PE engagement.

At the level of construct structure, the results showed that PAWR, SUV and SAVKSI are strongly associated with the HOC of individual interest, supporting its conceptualization as a coherent and multidimensional motivational disposition (22). This aligns with theoretical perspectives that frame interest as an integration of affective experiences, perceived value and knowledge-oriented tendencies (56, 83). However, despite the strength of this internal structure, the HOC exhibited only a small association with study engagement. This divergence suggests that while individual interest is conceptually well-formed, its aggregated representation may not fully capture the specific ways in which its components relate to engagement outcomes. In this sense, structural coherence does not necessarily translate into predictive strength when factors are combined into a single construct.

When examined at the dimensional level, a more differentiated pattern emerged. Among the three factors, only PAWR demonstrated a statistically significant association with study engagement. This finding should not be interpreted as evidence that affective interest is universally more important than utility- or attainment-based interest across all PE contexts. Rather, it suggests that the engagement relevance of individual interest is context-sensitive. The tripartite model identifies PAWR, SUV and SAVKSI as distinct but related factors of individual interest (22), while Self-Determination Theory helps explain why the affective factor may be more immediately connected to engagement. From this perspective, enjoyment, positive affect and willingness to reengage may energize students' participation because they are closely tied to intrinsic motivation, volitional involvement and felt satisfaction during movement-based learning (68, 84).

In an integrated PATH-Fit context involving individual and dual sports, PAWR may be especially proximal to engagement because students encounter the activity through direct bodily participation. These activities often involve repeated practice, visible skill execution, immediate feedback, manageable challenge and, in dual sport tasks, responsive interaction with an opponent. Such features may make students' affective responses more immediately relevant to vigor, dedication and absorption. In this sense, affective attraction may function as an entry point to engagement, not because it fully explains engagement, but because it captures the felt quality of participation that students experience before they evaluate the broader utility or personal significance of the activity.

In contrast, SUV and SAVKSI did not demonstrate statistically significant associations with study engagement in the dimensional model. Expectancy-Value Theory provides a useful explanation for this pattern because utility and attainment values tend to become engagement-relevant when learners perceive a clear connection between the activity and their goals, self-concept or future use (72, 85). In the present context, students may recognize that individual and dual sports support fitness, skill development, recreation or competence, but these meanings may remain too general or distal unless they are explicitly activated through instruction. Similarly, knowledge-seeking intentions may not immediately translate into engagement during activity sessions when the learning environment emphasizes performance execution, repeated practice and immediate participation.

Taken together, the tripartite model explains what dimensions constitute individual interest, SDT explains why affective enjoyment may be more immediately energizing, and EVT explains why utility- and attainment-oriented meanings may require stronger instructional scaffolding before becoming engagement-relevant. Thus, the non-significant findings for SUV and SAVKSI do not suggest that usefulness, personal importance or knowledge-seeking are irrelevant to PE. Rather, they indicate that these factors may operate as more distal or scaffold-dependent components of individual interest. Students may need explicit opportunities to connect individual and dual sports to lifelong physical activity, movement confidence, tactical understanding, recreational competence and personal development before these values become more strongly associated with engagement.

The comparison between the higher-order and dimensional models further highlights both methodological and conceptual implications. The higher-order model provides a holistic representation of individual interest (86), while the multidimensional nature of individual interest in physical education supports modeling the construct through distinct but related components (22). However, it may reduce predictive specificity by combining factors that are not equally associated with the outcome. In contrast, the dimensional model offers greater explanatory detail by identifying which components are more closely associated with engagement. This distinction reflects an important consideration in modeling motivational constructs, where the choice between aggregation and differentiation carries implications for both interpretation and explanatory power, as suggested in broader work on multidimensional constructs (87, 88). The present findings suggest that while higher-order modeling is valuable for conceptual clarity, dimensional analysis remains essential for capturing context-sensitive relationships and maintaining predictive specificity (88, 89), especially in physical education where motivational processes are shaped by task value and learning context (90). This approach avoids the “averaging out” effect where a global factor may obscure the unique variance of individual sub-dimensions (86). Therefore, the integrated PATH-Fit context is important because it clarifies the level at which the findings should be interpreted. The study does not compare individual and dual sports as separate modalities; rather, it examines students' overall experience of a course context where both activity formats are taught together. The findings should therefore be understood as preliminary evidence of how individual interest relates to engagement within this combined learning environment, not as evidence of differences between sport modalities.

Overall, these patterns suggest that affective components of interest may function as more proximal correlates of engagement, whereas value- and identity-related components may operate as more distal or context-dependent influences. This interpretation is compatible with situated EVT's emphasis on proximal decision processes (85) and with SDT's focus on the immediate energizing role of intrinsic motivation (68). Taken together, the findings suggest that individual interest should be understood both as a unified construct and as a set of distinct, interrelated factors whose relevance varies depending on context and level of analysis. By integrating higher-order modeling with dimensional analysis, the study offers a preliminary but more nuanced account of how individual interest may operate in a context-sensitive manner in PE. This approach clarifies the structure of the construct while also emphasizing that its relationship with engagement is neither uniform nor fixed, but contingent on how its components interact with the characteristics of the learning environment.

## Conclusion

This study examined the association between individual interest and study engagement within an integrated PE context involving individual and dual sports in Philippine HE. Results showed that individual interest functions as a coherent multidimensional construct, but its association with study engagement was small when modeled as a HOC. At the dimensional level, only the affective factor was significantly associated with engagement, while utility and attainment-oriented factors were not. These findings suggest that the relationship between individual interest and engagement is not uniform across its factors and may vary depending on how the construct is modeled and contextualized. However, given the small explanatory power of the models, the findings should be interpreted as preliminary evidence that affective attraction and willingness to reengage are relevant motivational correlates of study engagement, rather than as a comprehensive explanation of engagement in PE.

### Theoretical and practical contributions

This study contributes to the literature by offering a preliminary and context-sensitive refinement of the tripartite model of individual interest in PE. The findings do not introduce a new dimension of individual interest; rather, they clarify how the existing factors of the tripartite model may differ in their engagement relevance when examined within an integrated PATH-Fit context involving individual and dual sports. Specifically, the strong loadings of PAWR, SUV and SAVKSI on the HOC indicate that individual interest can be represented as a coherent multidimensional motivational construct. However, the small association between the HOC and study engagement suggests that structural coherence should not be equated with strong explanatory power. This qualifies the tripartite model by showing that its factors may form a unified construct while still contributing unequally to engagement outcomes. The dimensional findings further refine the model by indicating that PAWR, rather than SUV or SAVKSI, carried the clearest engagement-related signal in this context. Thus, the contribution of the study lies in demonstrating that the tripartite model may be theoretically useful not only for identifying the structure of individual interest, but also for examining how its affective, utility-based and attainment-oriented factors become more or less engagement-relevant depending on the immediacy and experiential demands of the PE learning context.

From a practical standpoint, the findings suggest that enhancing engagement in individual and dual sports requires more than presenting these activities as useful, skill-building or physically beneficial. Since PAWR emerged as the most salient factor, PE instruction should intentionally cultivate enjoyable, emotionally safe and reengagement-oriented movement experiences. For individual sport tasks, this may involve self-paced skill progressions, personal goal-setting, low-threat performance challenges and feedback that emphasizes improvement rather than comparison. For dual sport tasks, this may include structured partner rotation, modified games, cooperative-competitive drills and tactical mini-challenges that allow students to experience challenge without excessive performance pressure. At the same time, the non-significant findings for SUV and SAVKSI suggest that utility and attainment meanings should not be assumed to emerge automatically. Teachers may need to make these meanings explicit through short reflective prompts, progress tracking, links to lifelong physical activity, and discussions of how individual and dual sports develop transferable movement confidence, decision-making and recreational competence.

### Implications

The findings have implications for how individual and dual sports may be designed and taught in PE in the higher education. Since PAWR emerged as the only significant dimensional correlate of study engagement, teachers should not assume that students will become engaged simply because an activity is useful, skill-based or part of the required curriculum. Engagement may first need to be activated through the felt quality of participation. In practical terms, this means that individual and dual sports should be organized as enjoyable, emotionally safe and appropriately challenging learning experiences before expecting students to appreciate their broader utility or attainment value. Teachers may incorporate progressive task difficulty, modified rules, low-threat practice opportunities, personal goal-setting, peer-supported drills and feedback focused on improvement rather than comparison. These strategies may help students experience success, enjoyment and willingness to participate again, which are important affective conditions for sustaining engagement in sport-based PE.

At the curricular level, the findings suggest the need to move beyond traditional skill-and-drill approaches toward more contextualized, student-centered and meaning-oriented PE instruction. This direction aligns with competency-based curriculum thinking, where learning is organized around transferable capacities rather than isolated performance outcomes (91), and with physical literacy perspectives that emphasize motivation, confidence, physical competence, knowledge and understanding as foundations for lifelong engagement in movement (92). The findings also resonate with meaningful PE scholarship, which emphasizes that PE should provide experiences that students perceive as enjoyable, challenging, socially supportive, personally relevant and worth continuing beyond the lesson (93). In relation to the present findings, the salience of PAWR suggests that enjoyment, positive affect and willingness to reengage may serve as an affective foundation through which students become more open to developing competence, knowledge and continued participation.

This interpretation also suggests that cognitive and attainment-oriented values may not automatically translate into engagement unless students first experience movement as personally accessible, emotionally affirming and meaningful. The non-significant findings for SUV and SAVKSI do not mean that usefulness, knowledge or personal importance are irrelevant to PE. Rather, they indicate that these meanings may need to be deliberately scaffolded through instruction. Therefore, PE in the higher education may need to be reconceptualized not only as a venue for technical skill acquisition, but also as a learning space that supports student well-being, agency and movement confidence. Under this orientation, individual and dual sports instruction should help students experience choice, recognize personal progress, reflect on the relevance of movement to their lives, interact positively with peers, and understand how sport-based activities can be adapted for lifelong participation beyond formal PE classes.

### Limitations

Several limitations should be considered when interpreting the findings of this study. First, the study employed purposive sampling, which may limit the representativeness of the sample and restrict the generalizability of the results to broader populations of students in PE. Second, the cross-sectional design does not allow for the examination of changes in individual interest and study engagement over time, nor does it permit causal or temporal inferences among variables. Third, the use of self-report measures may introduce response-related biases, including social desirability and common method variance. Although anonymity, voluntary participation and VIF diagnostics were used to reduce and assess possible bias, these procedures do not fully eliminate the possibility of common-method-related effects. In particular, the VIF results should be understood only as a supplementary diagnostic rather than as a substitute for stronger research design features, such as temporal separation of measurements, multi-source data collection, or objective indicators of engagement. A further limitation concerns the unique curricular structure of PATH-Fit 4 in the Philippine HE setting. In this context, individual and dual sports are commonly delivered as part of one integrated PE course experience rather than as separate sport-specialized tracks. Thus, students' responses reflected their overall experience of a required PATH-Fit course involving both individual and dual sports, not isolated experiences in a single sport modality. This curricular arrangement is important because it reflects the way students actually encounter these activities in Philippine higher education PE. However, it also means that the findings should not be interpreted as evidence that the observed relationships operate identically in individual sports and dual sports when examined separately. Because the dataset did not include a grouping variable identifying whether each response was anchored specifically in an individual sport or a dual sport, moderation analysis or split-sample comparison by sport modality was not conducted. Finally, the study was conducted within a specific institutional and curricular context, which may limit the transferability of the findings to other PE programs, universities or national settings where individual and dual sports are organized differently. The small explanatory power observed in the models also suggests that other relevant variables not included in the present study may shape study engagement, including instructional practices, perceived competence, peer climate, teacher support, prior sport experience, assessment pressure and task design. Therefore, future studies may build on the present findings by examining these factors alongside individual interest and by designing sport-specific data collection procedures when separate analysis of individual and dual sports is intended.

### Future research considerations

Future research may extend the present findings by examining individual interest across a broader range of PE contexts and activity domains. Longitudinal designs may provide deeper insights into how the factors of individual interest develop and relate to engagement over time, especially as students accumulate repeated experiences with specific movement activities. Mixed methods research may also offer a more comprehensive understanding of how students experience, interpret and express affective, utility-based and attainment-oriented factors of interest within PE. In particular, qualitative data may help explain why some factors become more salient in certain activity contexts while others remain less visible in immediate engagement behaviors. Future studies may also examine individual and dual sports separately when the curriculum design and dataset allow such analysis. Researchers may ask students to identify the specific sport activity they are evaluating or collect separate responses for individual sport and dual sport units. This would make it possible to conduct multi-group analysis, moderation testing or sport-specific comparisons to determine whether the PAWR-engagement relationship differs by sport modality. Given the small explanatory power of the present models, future research may also incorporate additional predictors of study engagement, including perceived competence, autonomy support, peer interaction, learning climate, assessment pressure, movement confidence and prior sport experience. Experimental or intervention-based studies may further clarify how instructional strategies can strengthen the alignment between affective enjoyment, perceived utility, attainment value and engagement in sport-based PE.

### Positioning Filipino studies in the global literature

This study contributes to the global literature by providing context-specific evidence on individual interest and study engagement within Philippine HE, a setting that remains underrepresented in international scholarship. Beyond adding another empirical site, the study offers an epistemic acknowledgment of Filipino PE scholarship as a legitimate source of theoretical insight, not merely as a local application of models developed elsewhere. By demonstrating that the factors of individual interest are not equally associated with engagement and that their relevance varies across activity contexts, the findings challenge assumptions of uniformity in motivational processes. The study highlights the importance of examining motivation within localized educational settings, suggesting that insights from the Philippines can contribute to a more nuanced and globally relevant understanding of PE. In this sense, Filipino scholarship is positioned not as peripheral, context-bound or supplementary, but as an active contributor to broader theoretical and empirical discussions on motivation, engagement and movement-based learning. Such positioning is important because epistemic acknowledgment requires recognizing that knowledge generated from Philippine HE can refine, complicate and extend international conversations in PE, rather than simply confirm frameworks established in more frequently studied contexts.

## Data Availability

The raw data supporting the conclusions of this article will be made available by the authors, without undue reservation.
